# Assessing the potential of rural and urban private facilities in implementing child health interventions in Mukono district, central Uganda–a cross sectional study

**DOI:** 10.1186/s12913-016-1529-9

**Published:** 2016-07-15

**Authors:** Elizeus Rutebemberwa, Esther Buregyeya, Sham Lal, Sîan E. Clarke, Kristian S. Hansen, Pascal Magnussen, Philip LaRussa, Anthony K. Mbonye

**Affiliations:** Department of Health Policy, Planning and Management, Makerere University School of Public Health, Kampala, Uganda; Department of Disease Control and Environmental Health, Makerere University School of Public Health, Kampala, Uganda; Department of Disease Control, London School of Hygiene and Tropical Medicine, Keppel Street, London, WC1E 7HT UK; Department of Global Health and Development, London School of Hygiene and Tropical Medicine, 15-17 Tavistock Place, London, WC1H 9SH UK; Centre for Medical Parasitology, University of Copenhagen, Copenhagen, Denmark; Department of Pediatrics, College of Physicians & Surgeons, Columbia University, New York, USA; Ministry of Health, Kampala & School of Public Health-Makerere University, Box 7272, Kampala, Uganda

## Abstract

**Background:**

Private facilities are the first place of care seeking for many sick children. Involving these facilities in child health interventions may provide opportunities to improve child welfare. The objective of this study was to assess the potential of rural and urban private facilities in diagnostic capabilities, operations and human resource in the management of malaria, pneumonia and diarrhoea.

**Methods:**

A survey was conducted in pharmacies, private clinics and drug shops in Mukono district in October 2014. An assessment was done on availability of diagnostic equipment for malaria, record keeping, essential drugs for the treatment of malaria, pneumonia and diarrhoea; the sex, level of education, professional and in-service training of the persons found attending to patients in these facilities. A comparison was made between urban and rural facilities. Univariate and bivariate analysis was done.

**Results:**

A total of 241 private facilities were assessed with only 47 (19.5 %) being in rural areas. Compared to urban areas, rural private facilities were more likely to be drug shops (OR 2.80; 95 % CI 1.23–7.11), less likely to be registered (OR 0.31; 95 % CI 0.16–0.60), not have trained clinicians, less likely to have people with tertiary education (OR 0.34; 95 % CI 0.17–0.66) and less likely to have zinc tablets (OR 0.38; 95 % CI 0.19–0.78). In both urban and rural areas, there was low usage of stock cards and patient registers. About half of the facilities in both rural and urban areas attended to at least one sick child in the week prior to the interview.

**Conclusion:**

There were big gaps between rural and urban private facilities with rural ones having less trained personnel and less zinc tablets’ availability. In both rural and urban areas, record keeping was low. Child health interventions need to build capacity of private facilities with special focus on rural areas where child mortality is higher and capacity of facilities lower.

## Background

Private facilities play a significant role in the delivery of health services in low-and middle-income countries [1–3]. Private facilities include clinics, pharmacies or drug shops which are privately owned and where medical or non-medical personnel offer diagnosis and/or treatment for a fee. Private clinics and drugs shops are usually the first place of care seeking for children under five years of age [4] and it has been recommended that private facilities be engaged in health interventions [5]. Various initiatives have been conducted with private facilities in an effort to increase coverage and utilization of healthcare but their results are mixed with quality of care not being sustainable [6, 7]. However excluding private facilities from implementing health interventions sometimes limits the effects in the public sector [8]. There is need for more research into the understanding of how private facilities can be utilized in serving public health policy goals especially child health interventions [9].

The private health care facilities are not evenly distributed between the urban and rural areas with, the urban areas having a higher proportion [10]. The context, whether urban or rural, in which an intervention is implemented affects the way it impacts on health outcomes [11, 12]. This calls for a stratification of urban and rural areas when designing health interventions. It would also be critical to examine what differences exist between private facilities located in the urban areas vis-à-vis those in the rural areas so that when interventions are to be administered, the gaps that exist in the different areas are appropriately addressed and the opportunities identified and adequately utilized.

The objective of this analysis was to assess the rural–urban differences among private facilities in a district in Uganda with respect to the characteristics of diagnostic capabilities, operations, their attendants and the in-service training they have received in malaria, pneumonia and diarrhoea management.

## Methods

### Study area

The study was done in Mukono district in central Uganda. Mukono district headquarters is about 27 km east of the capital Kampala. The district had a projected mid-year population of 565,700 in 2013 [13] distributed between 13 sub-counties and one town council. Children below five years were estimated to constitute about 30 % and urbanization was estimated at 17 % [14]. There were 41 government health facilities of which three are Health Centre (HC) IVs, 15 HC IIIs and 23 HC IIs. There were also nine Non-Governmental facilities of which two are hospitals [14]. There are a number of private clinics and drug shops but some of them are unregistered and harder to reach [10].

Private facilities in this study area include drug shops, private clinics and pharmacies. Drug shops mainly sell drugs, are registered under trained health workers but sometimes the people left to attend to patients are untrained. Some drug shops also offer clinical assessment although this is not their main function and it may be restricted to only asking about symptoms. Private clinics provide both clinical assessment and drugs. As reported by Tawfik et al., the distinction between drug shops and private clinics is blurred as the two types of facilities have close similarity in practices [15]. Pharmacies are bigger units registered under pharmacists and are usually found in big towns. They sell drugs as wholesalers and retailers. They are the main sources of drugs for the drug shops and private clinics in the surrounding areas.

### Study design and population

This was a cross sectional study done in all the parishes of Mukono district in October 2014 that fulfilled the following criteria: the parish had to have a government health facility at least at the level of a Health Centre II which is the lowest level for facility based health care for the public sector. The parish also had to have a private facility like a drug shop, private clinic or pharmacy. There were supposed to be at least 200 households which figure was taken to indicate a potential sizable demand for health care. Out of the 84 parishes in Mukono district, 57 fulfilled the criteria and the study was done in all of them.

All the pharmacies, private clinics and drug shops, registered or unregistered, which consented to the interview, were included in the study. All the registered private facilities were interviewed. A few of the unregistered private facilities declined the interview. The numbers of those who declined could not be easily established as some were reported to have closed down. All the pharmacies were registered and were included in the study. Since this study included all the private facilities that were found in those parishes that fulfilled the criteria, the sample could be considered adequate to represent the district under study. The respondent in each private facility was the health care provider who was found on duty at the time of the visit. This study is part of the baseline for a study on referral of children from private to public facilities.

### Data collection

There were five interviewers with experience of collecting quantitative data for more than five years each, fluent in both English and the local language Luganda and sensitized on the objectives of the study and the tools. The tools, which were structured questionnaires, were piloted to see whether the questions were understood by the respondents and whether the tools were collecting the variables they were intended to collect. The interviews were conducted in English as all the respondents who work in these registered pharmacies, private clinics and drug shops know English. The interviews were conducted at a time when the provider would not be very busy to limit inconvenience to the private facility.

The variables being collected were: location–urban or rural, type of facility and registration status. For the attendant who was found in the private facility, variables collected were: sex, professional training received, highest level of education and any in-service training on malaria, pneumonia and diarrhoea. The variables collected on diagnostic ability included availability of thermometer, having a microscope and presence or absence of Rapid Diagnostic Tests (RDTs). An assessment of the presence of guidelines, patient registers, and availability of essential drugs as well as workload in terms of the number of children below five years seen at the facility was also collected. The essential drugs for malaria assessed were Chloroquine, Fansidar, Arthemether/Lumefantrine (Coartem also called Lumartem), Camoquine, Quinine and Metakelfin. For antibiotics, those assessed were: Amoxycillin, Trimethoprim/Sulphamethoxazole (Cotrimoxazole also called Septrin), Tetracycline, Gentamycin and Penicillin. For diarrhoea, the drugs assessed were zinc tablets and oral rehydration salt.

### Data management and analysis

Data was collected and reviewed periodically by the investigators. Data was double entered in Microsoft Access (Microsoft Inc., Redmond, Washington) and checked for variations, which were cross-checked with the raw data and corrected. Data was then transferred to Epi Info 7 and analysed. A line list was done to check for any gaps or inconsistencies. The data was then stratified into that from rural or urban areas. Urban areas were those areas which the Uganda government gazetted as urban and those not gazetted as urban were taken as rural. A comparison was made between the rural and urban private facilities with respect to the variables collected in terms of infrastructure at the facilities and the training of the person found attending to the patients. The outcome variables were categorical and a variable was considered present or absent. Univariate analysis was done to get the frequencies of the variables in each group. Bivariate analysis was done to get associations. Results were presented in tables using frequencies and associations. Those variables whose association *p*-value was less than 0.05 were taken to be significant. Where the numbers in the cells were small, a Fisher’s exact test was used to test for significance.

## Results

There were more private health facilities in the urban 80.5 % (194/241) compared to rural areas 19.5 % (47/241) (Table 1). Of the 47 rural facilities, there was only one pharmacy (2.1 %), 6 (12.8 %) private clinics and 40 (85.1 %) drug shops. Rural private facilities were more likely to be drug shops compared to urban facilities (OR 2.80; 95 % CI 1.23–7.11) and less likely to be private clinics (OR 0.39; 95 % CI 0.14–0.93). Rural facilities were less likely to be registered with the National Drug Authority (NDA) (OR 0.31; 95 % CI 0.16–0.60), did not have any health workers who were trained as clinicians such as doctors or clinical officers, were more likely to have nursing assistants or aides managing them (OR 3.43; 95 % CI 1.76–6.71), and were less likely to have people with tertiary education (OR 0.34; 95 % CI 0.17–0.66). Of the 47 rural facilities, 26 (55.3 %) were registered compared to 165/194 (85.9 %) in urban areas.

**Table 1:** Private facilities in the rural and urban areas of Mukono District, their attendants and diagnostic capabilities

Variable^b^	Rural (*n* = 47)	Urban (*n* = 194)	OR (95 % CI)	*p*-value
Types				
Drug shop	40 (85.1)	130 (67.0)	2.80 (1.23–7.11)	0.009
Private clinic	6 (12.8)	53(27.3)	0.39 (0.14–0.93)	0.017
Pharmacy	1(2.1)	11(5.7)	0.36 (0.01–2.61)	0.530
Registration				
Facility registration	26 (55.3)	165 (85.9)	0.22 (0.10–0.47)	<0.001
Registration with NDA^a^	16 (34.0)	150 (62.2)	0.31 (0.16–0.60)	<0.001
Sex of attendants				
Females	43 (91.5)	146 (75.3)	3.53 (1.19–14.20)	<0.001*
Professions of persons managing the facilities				
Doctor/clinical officer	0 (0.0)	23 (11.9)	-	-
Registered nurse/midwife	1 (2.1)	12 (6.2)	0.33 (0.01–2.34)	0.239*
Enrolled nurse/midwife	12 (25.5)	63 (32.5)	0.71 (0.35–1.47)	0.183
Nursing assistant/aide	31(66.0)	70 (36.1)	3.43 (1.76–6.71)	<0.001
Other	3 (6.4)	26 (13.4)	0.44 (0.09–1.54)	0.139*
Highest level of education of persons found in the facilities				
Secondary	31 (66.0)	71(36.6)	3.56 (1.72–6.56)	<0.001
Tertiary (certificate/diploma)	16 (34.0)	117 (60.3)	0.34 (0.17–0.66)	<0.001
University	0 (0)	6 (3.1)	-	-
Diagnostic capacity				
Availability of a thermometer	44 (93.6)	185 (95.4)	0.71 (0.17–4.27)	0.425
Having a microscope	5 (10.6)	46 (23.7)	0.38 (0.11–1.05)	0.077
Presence of RDTs at the facility	21 (46.7)	115 (59.3)	0.55 (0.29–1.06)	0.057
Having a copy of the malaria treatment guidelines	8 (17.0)	53 (27.3)	0.55 (0.21–1.29)	0.100

* Fisher’s exact test, ^a^ NDA = National Drug Authority

^b^Each of the variable is tabulated against the other variables together

On stratification across the different types of facilities, 31/47 (66.0 %) of the facilities in rural areas had attendants whose highest level of education was secondary school while the rest were of higher level education. However of these 27/31 (87.1 %) were in drug shops. A total of 33/47 (70.2 %) of the rural facilities had no patient register of whom 32/33 (97.0 %) were drug shops. With regard to training, 31/47 (66.0 %) rural facilities were attended to by nursing assistants or nursing aides who have very little training in clinical work. Of these 27/31 (87.1 %) were in drug shops. In terms of stock cards, only five facilities in rural areas had stock cards. A total of 31/47 (66.0 %) of the rural facilities had zinc tablets. However of these 27/31 (87.1 %) were drug shops. In urban areas, 162/194 (83.5 %) private facilities had zinc tablets. Bivariate analysis by facility type could not be done because of the very small numbers for the private clinics and pharmacies. With only one pharmacy and six private clinics in the rural areas, the numbers in certain cells were so small for meaningful analysis.

Although the urban facilities had a higher percentage with microscopes and Rapid Diagnostic Tests (RDTs) than the rural facilities, the differences were not statistically significant. In both urban and rural areas, private facilities did not have microscopes with only 46/194 (23.7 %) facilities in urban areas having a microscope and in rural areas only 5/47 (10.6 %) had a microscope. In the study, we never tested whether these microscopes were used. With the general lack of qualified personnel in villages, it is possible that the utilization of these microscopes was not optimal.

All facilities in the urban and rural areas had antimalarials. However for the first line antimalarial Athemether/Lumefatrine, only two drug shops and one private clinic (3/47, 6.38 %) reported not to have them in rural areas while in urban areas it was 3/194 (1.55 %). The first line antibiotic for pneumonia is amoxicillin and of the 47 private facilities in the rural areas, 43 (91.3 %) stocked it while in urban areas it was 180/194 (92.8 %).

There was no significant difference between the urban and rural facilities in terms of presence or absence of patient registers, types of drugs sold, presence or absence of stock cards, giving patients injections, places where they purchase drugs or the proportion of children seen with severe illness except that rural facilities were less likely to have zinc tablets in stock compared to urban facilities (OR 0.38; 95 % CI 0.19–0.78) as shown in Table 2. More than half of the urban facilities operate without patient registers 115/194 (59.3 %) while in the rural areas it was more than seventy percent 33/47 (70.2 %). In both urban and rural areas, there was low usage of stock cards with only 5/47 (10.6 %) rural facilities having them compared to 44/194 (22.7 %) urban facilities. When asked whether the facility had attended to a child with severe illness in the previous week, almost half of the facilities in rural areas 23/47 (48.9 %) and a slightly higher percentage in urban areas 102/194 (52.6 %) indicated they had seen such a child.

**Table 2:** Operations of private rural and urban facilities in Mukono District

Variable	Rural (*n* = 47)	Urban (*n* = 194)	OR (95 % CI)	*p*-value
Presence of a patient register				
Having a patient register present	14 (29.8)	79 (40.7)	0.62 (0.31–1.23)	0.085
Drugs being sold				
Antimalarials	47 (100)	194 (100)	-	-
Antibiotics	46 (97.9)	184 (94.8)	2.50 (0.34–110.82)	0.331*
Zinc tablets	31(66.0)	162 (83.5)	0.38 (0.19–0.78)	0.007
ORS	44 (93.6)	190 (97.9)	0.31 (0.05–2.20)	0.272*
Presence of stock cards				
Has stock control cards	5 (10.6)	44 (22.7)	0.41 (0.12–1.12)	0.101
Injections given to patients	30 (63.8)	120 (62.2)	1.08 (0.56–2.11)	0.802
Where drugs are purchased				
Pharmacy	47 (100)	193 (99.5)	-	-
Health units	7 (14.9)	28 (14.4)	1.04 (0.42–2.55)	0.936
Open markets	6 (12.8)	23 (11.9)	1.09 (0.34–2.98)	0.938*
Children with severe illness seen				
Having seen at least one child with severe illness in the previous week	23 (48.9)	102 (52.6)	0.86 (0.42–1.66)	0.654

* Fisher’s exact test

The results in Table 3 show the in-service training status for persons found attending to patients in the private facilities. Training on malaria management had been given to more than half of facility staff in rural 26/47 (55.3 %) and 108/194 (55.7 %) in urban areas. Training on management of diarrhea had been given to 29.8 % (14/47) of facility staff in rural areas compared to 36.6 % (71/194) in urban areas. Training on pneumonia management had been given to 6 (12.8 %) private providers in rural areas compared to 24 (12.4 %) in urban areas. None of the differences in training between the health workers in the rural and urban private facilities were significant.

**Table 3:** In-service training received by persons attending to patients at private facilities

Variable	Rural (*n* = 47)	Urban (*n* = 194)	OR (95 % CI)	*p*-value
Having attended training workshop on malaria management	26 (55.3)	108 (55.7)	0.99 (0.52–1.87)	0.965
Having attended training on management of pneumonia	6 (12.8 %)	24 (12.4)	1.04 (0.33–2.83)	0.863*
Did training to cover antibiotics	5 (10.6)	20 (10.3)	1.04 (0.29–3.07)	0.841*
Did training to cover IMCI guidelines	6 (12.8)	20 (10.3)	1.27 (0.39–3.56)	0.822*
Training on management of diarrhoea	14 (29.8)	71(36.6)	0.74 (0.37–1.47)	0.381
Training covered microscopy	1(2.1)	13 (6.7)	0.30 (0.01–2.12)	0.393*
Training covered Rapid Diagnostic Tests	7 (14.9)	28 (14.4)	1.04 (0.36–2.66)	0.881*
Training covered Artemesinin Combination Therapies	6 (12.8)	23 (11.9)	1.09 (0.34–2.98)	0.938

* Fisher’s exact test

## Discussion

Differences existed between rural and urban private facilities. First, there were fewer private facilities in rural areas compared to urban areas. Secondly, workers attending to patients in private facilities in rural areas were more likely to have attained lower education standards and were less trained in clinical work than their counterparts in urban areas. Thirdly, private facilities in rural areas had less diagnostic equipment compared to those in urban areas although the availability of microscopes and Rapid Diagnostic Tests (RDTs) was generally low in both urban and rural areas. In addition, in terms of drug availability, rural areas were more likely to lack zinc tablets than do the urban private facilities. Fourthly, patient registers and stock cards were more likely to be lacking in rural areas than urban facilities. Finally, there was low in-service training in the management of malaria, pneumonia and diarrhoea for health workers in private facilities in both urban and rural areas.

Similar to the study that was done in Iganga district in eastern Uganda [16], there were more private health facilities in urban areas in Mukono district than the rural areas. This has implications on policy implementation of involving private facilities in health interventions. The urban population in Uganda is about 18 % of the total population [17] and yet has 80 % of the private facilities. If the implementation includes whichever private provider they can access within the district without taking into consideration the locality of the facility whether urban or rural, such an intervention will just exacerbate the inequality of accessing health services as more of these interventions will more likely be situated in towns rather than rural areas where access to health services is low.

Health workers in the rural private facilities were likely to be less trained and with lower professional qualifications than those in urban areas. Rural areas are likely to have drug shops rather than clinics compared to urban areas. Another study done in Kampala indicated that most people in drug shops have low knowledge on the signs and symptoms of childhood illnesses like pneumonia [18]. This has implications on the level of care one would get when one accesses the drug shops. Whereas private clinics routinely conduct clinical assessment before distributing the drugs, drug shops give out drugs to clients on request with or without prescription. In terms of utilizing these drug shops for health interventions, it is important that the professional education of these workers be put into consideration otherwise, an intervention could be relegated to a person least prepared to handle it especially if it requires clinical capabilities. In such a case, the requirement to have clinical training could be a pre-requisite for handling clinical work or the workers need to undergo clinical training before they are given more complex clinical responsibilities. An analysis across different countries has noted poor diarrhoea management practices in rural areas among non-governmental providers, a likely outcome of the low training the providers in rural private facilities may be having [19].

Our study indicated a low proportion of private facilities with diagnostic facilities like microscopes and RDTs. Various studies have indicated that patients seek care from public health facilities because of better diagnostic capacity than private facilities [3, 20]. Some of the interventions that have been suggested to be done in private facilities include the management of malaria [5]. However with the decrease in the prevalence of malaria in some countries, it is recommended that laboratory investigations be carried out before giving people treatment [21]. Before interventions like provision of Arthemisinin Combination Therapies (ACTs) are rolled out into private facilities, it is critical that the capacity of each private health facility to diagnose malaria be assessed to avoid wastage of drugs and increase in drug resistance.

The study found that majority of the private facilities did not have a patient register nor stock cards. Without these basic tools, it is difficult for the health facility to assess its performance as well as account for the supplies utilized in the health intervention. It is critical therefore that when interventions are being rolled out, training and enforcement of the use of patients registers and stock cards be done so that when an assessment is made on the level of utilization of a service, an appropriate value is obtained.

It was found that very few health workers in private facilities especially those in rural areas had attended various types of in-service training. Lack of in-service training to private providers deprives them of the opportunities of getting up-to-date with the new treatment guidelines and this exposes the patients being treated in these facilities to inappropriate treatment. Secondly, in the event that an intervention is envisaged for implementation in private facilities, prior training in the current clinical practices would need to be considered rather than presuming that the private providers are up-to-date.

Rural facilities were more likely not to have zinc tablets compared to urban facilities. The reason behind this observation is not clear. It is possible that the awareness of zinc tablets as a remedy for diarrhea was not wide spread among the people that lived in rural areas in which case they would not demand for it from the private facilities they go to for treatment. A study from Nigeria indicated that almost half of the caretakers could indicate the dosage of zinc in 2011 months after being trained on the treatment of diarrhea [22]. This was in contrast to oral rehydration salt which was already having a high level of awareness as far back as 1997 [23]. An assessment on the knowledge about the role of zinc tablets in the management of diarrhea needs to be done in rural areas and if found low, more sensitization would need to be done to increase its demand.

This study had limitations. The persons interviewed were those who were found attending to patients. It was possible that these were not the most highly qualified providers at that facility. However, from the perspective that these were the providers whom the patients would encounter once they came to seek treatment from the facility, it was a more real life situation as to what possible providers are available to serve clients seeking for care. The second limitation was that whereas the categorization was between urban and rural private facilities, it is possible that an urban private facility is more accessible than a rural facility depending on the opening hours that the facility provides and the availability of means of transport to that urban facility. That not-with-standing, the extra transport costs sometimes are an impediment to accessing health care and the presence of quality providing private facilities in rural areas would reduce the transport costs that could be incurred while seeking care in urban areas. Another limitation is that there were very few facilities from rural areas compared to the ones in urban areas. A similar result was found in a study in another district [16]. As all the facilities whose attendants consented to the study registered or not were interviewed, most of the rural private providers were captured in the sample. Limitations in sample size have hampered a more stratified analysis according to facility types. The results may only be generalizable to areas with similar distribution of private facilities and operational capabilities.

Whereas private providers present an opportunity to improve access to child health interventions like management of malaria, pneumonia and diarrhoea, there needs to be prior training for the specific type of intervention to the providers in these private facilities. More training is needed on recording and documentation using patients’ registers and stock cards. It is to be noted however, that rural private providers lag behind their urban counterparts in terms of professional qualifications and diagnostic equipment. In case of prioritization therefore, rural private facilities need more support.

## Conclusions

Private providers are more concentrated in the urban areas than the rural areas. Compared to urban areas, private facilities in rural areas had less trained personnel and less zinc tablets’ availability. There was a general lack of microscopes and RDTs in both urban and rural private facilities. The majority of private providers in both the rural and urban areas did not use stock cards nor patients’ registers. It is critical that before private facilities are involved in child health interventions, an assessment is done and capacity building is conducted in order to equip these private facilities with the human resources, the infrastructure and equipment necessary to handle the child interventions.
